# Supplementary material for: Synthesis and Anticancer Activity of Indole-Functionalized Derivatives of Betulin
Source: Pharmaceutics. 2022 Nov 4;14(11):2372. doi: 10.3390/pharmaceutics14112372 (PMC9694481; doi:10.3390/pharmaceutics14112372)
Supplement: Supplementary file 1 [file pharmaceutics-14-02372-s001.zip › pharmaceutics-1961681-supplementary.pdf]

Supplementary Materials

Synthesis and anticancer activity of indole-functionalized derivatives of betulin

Zuzanna Rzepka [1,*], Ewa Bębenek [2], Elwira Chrobak [2] and Dorota Wrześniok [1,*]

[1] Department of Pharmaceutical Chemistry, Faculty of Pharmaceutical Sciences in Sosnowiec, Medical University of Silesia in Katowice, 4 Jagiellońska Str., 41-200 Sosnowiec, Poland

[2] Department of Organic Chemistry, Faculty of Pharmaceutical Sciences in Sosnowiec, Medical University of Silesia in Katowice, 4 Jagiellońska Str., 41-200 Sosnowiec, Poland

* Correspondence: zrzepka@sum.edu.pl (ZR), dwrzesniok@sum.edu.pl (DW)

LIST OF CONTENTS

Fig. S1. ^{1}H-NMR spectrum for EB355A (600 MHz, $CDCl_3$)

Fig. S2. ^{13}C-NMR spectrum for EB355A (150 MHz, $CDCl_3$)

Fig. S3. HRMS spectrum for EB355A

Fig. S4. ^{1}H-NMR spectrum for EB365 (600 MHz, $CDCl_3$)

Fig. S5. ^{13}C-NMR spectrum for EB365 (150 MHz, $CDCl_3$)

Fig. S6. HRMS spectrum for EB365

Table S1. Prediction of the ADMET profile of betulin based on computer calculations. **Table S2.** The theoretical values of lipophilicity for betulin, betulinic acid, EB355A and EB365

Fig. S1. ^{1}H-NMR spectrum for EB355A (600 MHz, $CDCl_3$)

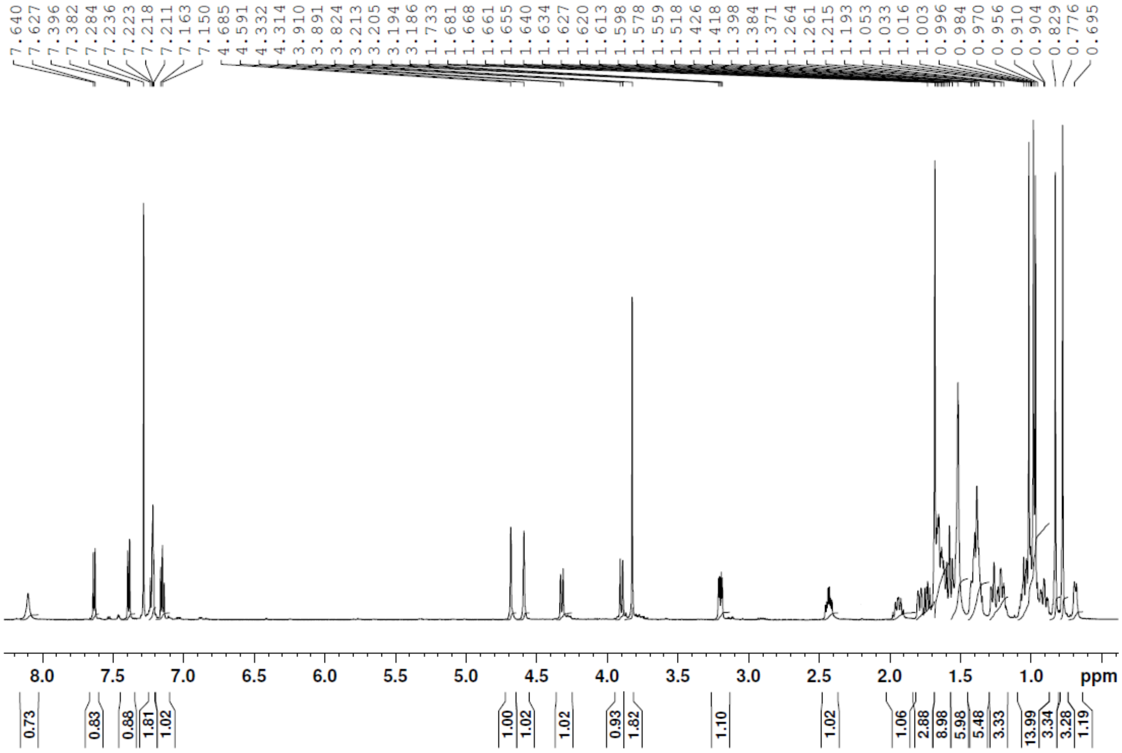

Fig. S2. ^{13}C-NMR spectrum for EB355A (150 MHz, $CDCl_3$)

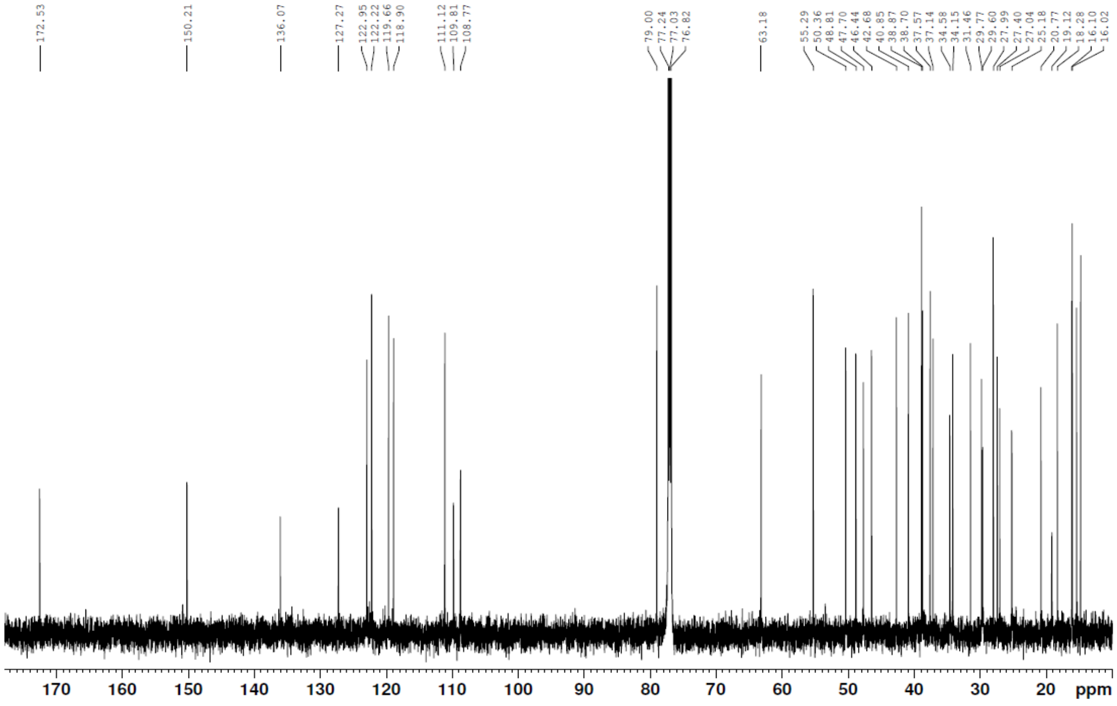

Fig. S3. HRMS spectrum for EB355A

Analysis Name	D:\Data\EB 355A		
Method	APCI_low_ mass_negative.m	Operator	KM
Sample Name	1-tolil	Instrument	impact II 1825265.10082
Comment			

Acquisition Parameter

Source Type	APCI	Ion Polarity	Negative	Set Nebulizer	2.0 Bar
Focus	Active	Set Capillary	4000 V	Set Dry Heater	200 °C
Scan Begin	100 m/z	Set End Plate Offset	-500 V	Set Dry Gas	4.0 l/min
Scan End	1600 m/z	Set Charging Voltage	2000 V	Set Divert Valve	Source
		Set Corona	15000 nA	Set APCI Heater	450 °C

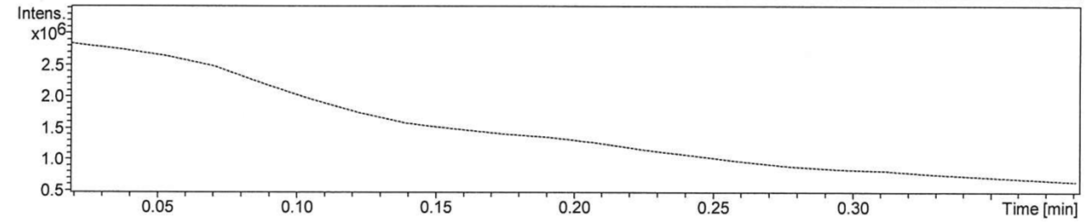

-MS, 0.0-0.2min #2-10

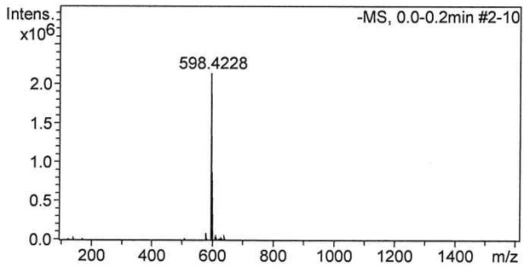

#	m/z	Res.	S/N	I	I %	FWHM
1	598.4228	19628	52253.3	2138804	100.0	0.0305

Fig. S4. ^{1}H-NMR spectrum for EB365 (600 MHz, $CDCl_3$)

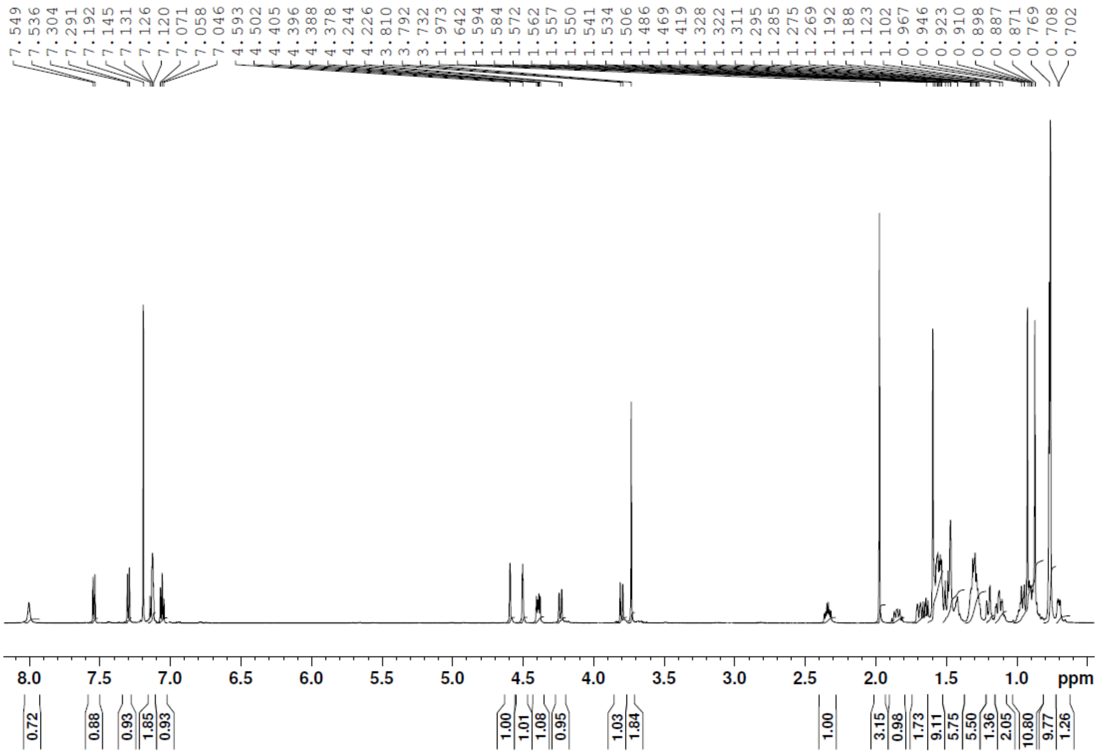

Fig. S5. ^{13}C-NMR spectrum for EB365 (150 MHz, $CDCl_3$)

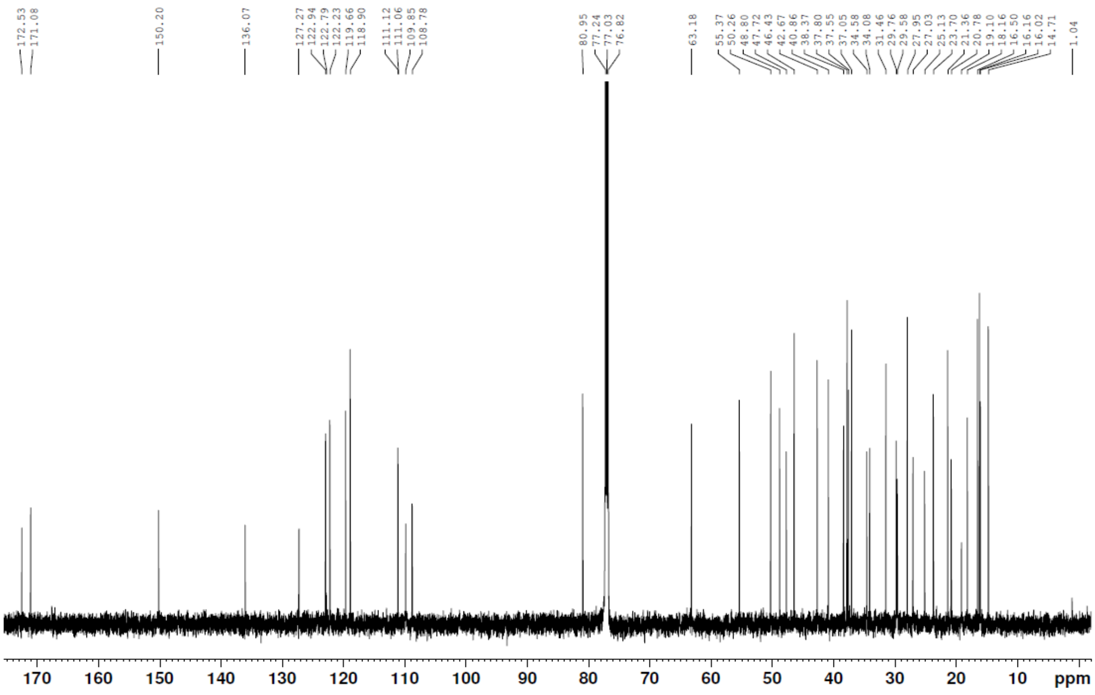

Fig. S6. HRMS spectrum for EB365

Analysis Name	D:\Data\EB 365		
Method	APCI_low_ mass_negative.m	Operator	KM
Sample Name	1-tolil	Instrument	impact II 1825265.10082
Comment			

Acquisition Parameter

Source Type	APCI	Ion Polarity	Negative	Set Nebulizer	2.0 Bar
Focus	Active	Set Capillary	4000 V	Set Dry Heater	200 °C
Scan Begin	100 m/z	Set End Plate Offset	-500 V	Set Dry Gas	4.0 l/min
Scan End	1600 m/z	Set Charging Voltage	2000 V	Set Divert Valve	Source
		Set Corona	15000 nA	Set APCI Heater	450 °C

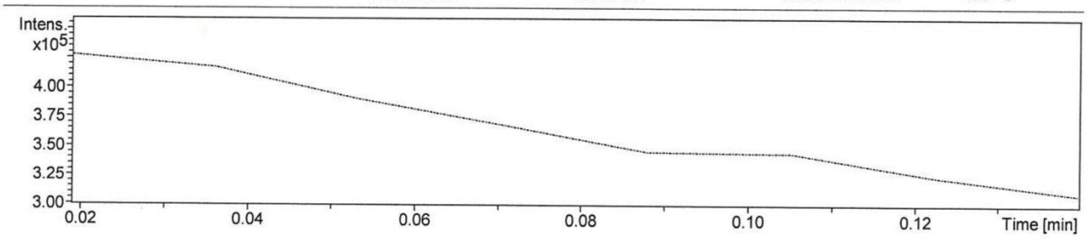

-MS, 0.0-0.1min #2-4

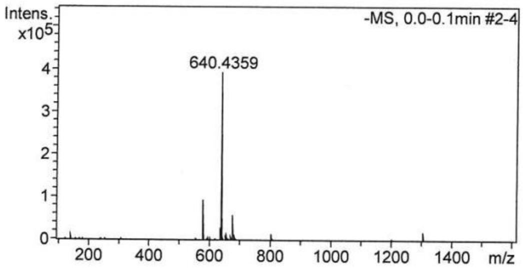

#	m/z	Res.	S/N	I	I %	FWHM
1	640.4359	13145	11385.9	392594	100.0	0.0487

Table S1. Prediction of the ADMET profile of betulin based on computer calculations.

Parameter	Betulin	
	Value	Probability
**Absorption**		
Human intestinal absorption (HIA)	+	0.9884
Caco-2 permeability	-	0.5542
Human oral bioavailability	-	0.5857
**Distribution**		
Subcellular localization	Lysosomes	0.4831
Blood brain barrier (BBB) permeability	-	0.4831
Organic Anion-Transporting Polypeptide (OATP) inhibitors:		
OATP 2B1	-	0.7184
OATP 1B1	+	0.9413
OATP 1B3	+	0.9480
Multidrug And Toxin Extrusion Transporter 1 (MATE1)	-	1.0000
Organic Cation Transport Protein 2 (OCT2) inhibitor	-	0.6385
Bile Salt Export Pump (BSEP) inhibitor	+	0.6370
P-glycoprotein inhibitor	-	0.8836
P-glycoprotein substrate	-	0.7347
**Metabolism**		
Cytochrome P450:		
CYP450 3A4 substrate	+	0.6751
CYP450 2C9 substrate	-	0.6284
CYP450 2D6 substrate	-	0.7222
CYP450 3A4 inhibition	-	0.8309
CYP450 2C9 inhibition	-	0.9071
CYP450 2C19 inhibition	-	0.9026
CYP450 2D6 inhibition	-	0.9297
CYP450 1A2 inhibition	-	0.8309
CYP inhibitory promiscuity	-	0.6441
**Toxicity**		
Carcinogenicity	-	0.9143
Eye corrosion	-	0.9927
Eye irritation	-	0.9265
Ames mutagenesis	-	0.6800
Hepatotoxicity	+	0.5250
Nephrotoxicity	-	0.9145
Acute Oral Toxicity	III	0.6406

Table S2. The theoretical values of lipophilicity for betulin, betulinic acid, EB355A and EB365

Compound	Log $P_{o/w}$	
	WLOGP	MLOGP
Betulin	7.00	6.00
Betulinic acid	7.09	5.82
EB355A	9.27	6.61
EB365	9.84	6.85
